# The diagnostic value of interleukin 35 as a septic biomarker: A meta-analysis

**DOI:** 10.3389/fmed.2022.999892

**Published:** 2022-11-10

**Authors:** Yuanhui Hu, Dongling Tang, Pingan Zhang

**Affiliations:** Department of Laboratory Medicine, Renmin Hospital of Wuhan University, Wuhan, China

**Keywords:** sepsis, biomarker, interleukin 35 (IL-35), meta-analysis, diagnosis

## Abstract

**Background:**

There is growing evidence that interleukin 35 (IL-35) represents a potential diagnostic biomarker for sepsis. The purpose of this meta-analysis was to evaluate the overall diagnostic accuracy of IL-35 in sepsis.

**Materials and methods:**

From October 1998 to May 2022, set retrieval standards were used to search literature Databases. Each included study was assessed diagnostic accuracy study quality assessment tool. Two researchers independently extracted the data and research features. If there are differences, the issue will be resolved by mutual agreement. Meta-disc and Stata software were utilized to calculate combined sensitivity, specificity, and summary diagnostic odds ratio (SDOR), *I*^2^, or Cochrane Q in order to detection for heterogeneity, and meta-regression was performed to figure out the cause of heterogeneity. Utilizing funnel plots, we tested for publication bias.

**Results:**

In this meta-analysis, eight publications were included. The combined sensitivity, specificity, and DOR were 0.87 (95% CI, 0.77–0.93), 0.73 (95% CI, 0.60–0.83), and 18.26 (95% CI, 9.70–34.37), respectively. In addition, 0.88 (95% CI, 0.84–0.90) was the area under the summary receiver operating characteristic curve. In the heterogeneity analysis, the sensitivity of comprehensive *I*^2^ statistic was 84.38, and the specificity was 87.82. Deeks’ funnel plot showed no publication bias in this meta-analysis (*P* = 0.17). A meta-analysis revealed that IL-35 has a modest sensitivity (AUC = 0.88) for diagnosing sepsis. We also compared the diagnostic accuracy of IL-35 and procalcitonin (PCT), and our results showed that the diagnostic accuracy parameters for IL-35 were significantly higher than those for PCT.

**Conclusion:**

Interleukin 35 is a valuable biomarker for the early detection of sepsis. However, the data should be combined with clinical symptoms, signs, and laboratory and microbiological findings.

## Introduction

Sepsis is an illness that may rapidly lead to death, resulting from an unbalanced body‘s immune responses to infections (1). In 2017, there were 48.9 million septic patients with high mortality of 22% and sepsis-related fatalities are 11 million, or 19.7% of all deaths globally (2). Early diagnosis of sepsis is the determining factor in patient outcome (3). The clinic doctor needs a diagnostic biomarker with high sensitivity and specificity to assist in prompt treatment. However, numerous biomarkers fail to meet the strict requirements for diagnostic and therapeutic use, and no biomarkers are currently suggested by sepsis recommendations (4). In addition, the time-consuming blood culture, which was long regarded as the primary diagnostic criterion, has been eclipsed by the prevalence of false-negative findings. And changes in cytokine mediators could be a helpful sepsis diagnosis or monitoring tool that might lead to early therapy and improved sepsis survival (5).

Interleukin 35 (IL-35), a heterodimer, has been seen as a novel cytokine, with other IL-12, IL-23, and IL-27 belonging to the IL-12 family, exerts significant roles in regulating host immunity (6). IL-35, unlike IL-12, IL-23, and IL-27 which promote inflammatory responses, can play substantial parts in suppressing immune reactions, secreted by regulatory monocytes, dendritic cells, T cells (Tregs), and macrophages (7, 8). It has been shown that IL-35 has several significant roles, including antioxidant, antiapoptotic, and anti-inflammatory properties. By decreasing the inflammatory response caused by LPS, IL-35 has been shown to protect against acute lung and kidney damage (9, 10). By reducing inflammation, apoptosis, and fibrosis, IL-35 ameliorates myocardial damage (11). Additionally, IL-35 reduces diabetic neuropathy pain through the JNK pathway and anti-apoptosis in the anti-inflammatory response (12). By activating the STAT1 and STAT4 signaling pathways, IL-35 has anti-inflammatory and antiapoptotic effects on LPS-induced endothelial cell activation, according to Li et al. (13).

In recent years, patients with sepsis shock and sepsis exhibited elevated IL-35 levels (14). These studies showed that IL-35 might be an early diagnostic biomarker for sepsis. Nowadays, the use of IL-35 has been reported in some clinically diagnostic studies for sepsis. Clinicians caring for patients with sepsis need to comprehensively evaluate the utility of IL-35 as a biomarker to provide information for evidence-based practice.

The diagnosis of sepsis is still regarded as challenging for both clinicians and the laboratory due to non-specific clinical presentations, so the current study examined IL-35 and compared the serum levels of procalcitonin (PCT) in sepsis to assess the most useful marker or markers regarding this issue (15). This meta-analysis will integrate existing knowledge and assess the utility of IL-35 as a diagnostic biomarker in patients with sepsis.

## Materials and methods

The Preferred Reporting Items for Systematic Reviews and Meta-analysis were used to perform this meta-analysis (23) and the Cochrane Handbook for Systematic Reviews of Interventions. Because all of the analyses were based on previously published research, there was no need for ethical approval or patient permission.

### Literature search

The related literature in these databases (EMBASE, the Cochrane Library, VIP, CNKI, WanFang Database, PubMed, and Web of Science) were systematically searched. According to relevant research reports, IL-35 was a useful biomarker to distinguish between septic and non-septic patients, published before May 12, 2022. Search keywords were as follows: a combination of “IL-35” and “sepsis.” In the process of literature collection, there are no language types or publication restrictions. We have also manually searched for printed articles, reviews, and references in the study. All related articles had been published.

### Criteria of selection

Case-control studies that employed IL-35 to distinguish between patients with sepsis and those without the disease were included in the meta-analysis. Thus, the case-control study afforded the chance to create two intricate two-by-two contingency tables. These were the criteria used to disqualify research from consideration: (1) case reports, meta-analyses, letters, correspondences, editorials, reviews, and animal studies; (2) missing or insufficient data.

### Data extraction

Using a two-level mixed logistic regression model with independent binomial distributions for the true positives and true negatives conditional on the sensitivity and specificity in each study, as well as a bivariate normal model for the logit transforms of sensitivity and specificity across studies, we were able to obtain pooled estimates of sensitivity and specificity. The study data were extracted independently by two reviewers, extracting the following information: first author names, country of study, year of publication, study design, number of cases and controls, Cutoff of IL-35, the type of sample, the detecting method, data of meta-analysis, and population type. Each research investigation’s degree of methodological rigor was assessed using the QUADAS-2 instrument (24).

### Statistical analysis

The diagnostic accuracy of IL-35 in sepsis was determined by combining the specificity, sensitivity, negative likelihood ratio (NLR), specificity, positive likelihood ratio (PLR), and diagnostic odds ratio (DOR). Next, the Symmetrical summary receiver operator curve (SROC) curve was constructed to illustrate our results. We used Meta-disc 1.4 in conjunction with Stata 17.0 in order to compute the area under the curve (AUC) and 95% credible interval (CI). A Fagan plot was created to display diagnostic performance graphically. Calculating the Spearman rank correlation coefficient (ρ), a non-parametric test that quantifies the degree of connection between sensitivity and specificity was used to examine the existence of threshold effect, along with the Spearman correlation coefficient in Meta-DiSc (1.4), and a *P*-value of 0.05 or below indicates a meaningful threshold influence. We characterized the heterogeneity of the meta-analysis using the *I*^2^ statistic. *I*^2^ levels below 50% are seen as being suggestive of significant heterogeneity. Using meta-regression and sensitivity analyses, the possible sources of heterogeneity were then examined [the population (adults/neonates), sample (serum/plasm), setting (ICU/other), severity of sepsis (severe sepsis or septic shock/other), site (celiac infection/other), diagnostic reference (sepsis-3/other), country (China and Iran), male (%) (≥55/<55), total number of samples (≥100/<100), and cutoff (≥50/<50 pg/ml)].

## Results

### Study characteristics and data extraction

**FIGURE 1:**
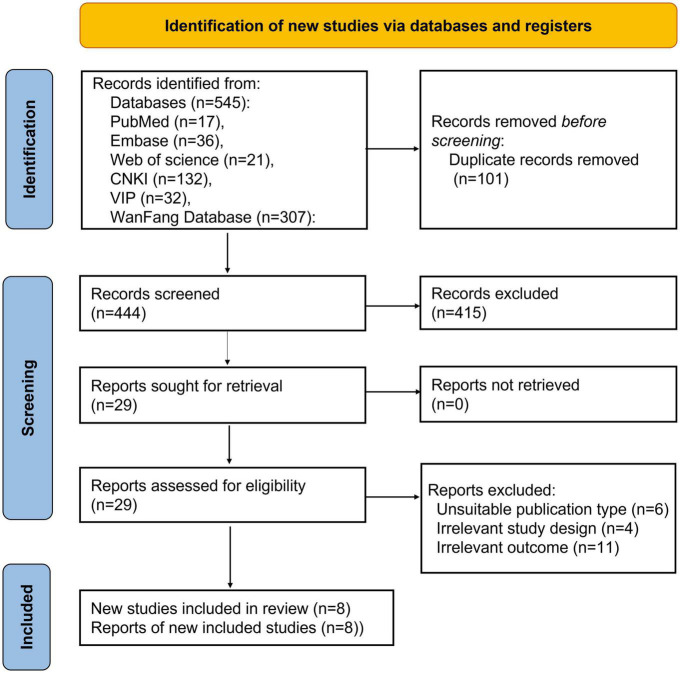
Flow chart of eight studies collection.

Five hundred forty-five articles were collected in the initial search from the database. Following carefully examining the titles and abstracts, we excluded 101 articles. We reviewed 29 articles and deleted 21 after carefully evaluating the full text. Finally, eight pieces (5, 16–22), were included in this analysis. Figure 1 shows the screening process in the flow chart. Thus, eight studies, comprising 599 patients with sepsis and 466 in control groups, were conducted in Iran and China, respectively. The patient groups included were adults and neonates. Table 1 offers a summary of the demographic data relevant to the aforementioned eight studies.

**TABLE 1:** Features of the research that were included.

References	Population	Country	Design	Sample	Method	Cutoff	Male (%)	Setting	Sample size				Total number	TP	FP	FN	TN	Diagnostic power		
															
									Case	Number	Control	Number						Sen (%)	Spe (%)	AUC
Du et al. (16)	Neonates	China	P	Serum	CLEIA	31.7 pg/ml	58.2	ICU	Sepsis	79	Non-infection	78	157	62	26	17	52	78.48	66.67	0.756
Xiaofei et al. (17)	Adults	China	P	Plasm	CLEIA	26.2 pg/ml	52	Gastrointestinal Surgery	Sepsis	68	LI	60	128	57	6	11	54	83.8	90	0.93
Zhixia et al. (18)	Adults	China	P	Serum	CLEIA	105.60 ug/l	55.8	ICU	Sepsis	120	HC	30	150	88	7	32	23	73.25	76.5	0.652
Yucang et al. (19)	Adults	China	P	Serum	CLEIA	41.97 pg/ml	45.9	ICU	Sepsis	74	HC	60	134	70	24	4	36	94	60	0.76
Saboute et al. (5)	Neonates	Iran	P	Serum	CLEIA	8.05 pg/ml	77.5	ICU	Sepsis	40	Non-septic	40	80	39	27	1	13	97	32	0.895
Junmin (20)	Adults	China	R	Serum	CLEIA	10.99 pg/ml	55	ICU	Sepsis	40	HC	20	60	38	4	2	16	95	80	0.834
Zhiyong (21)	Adults	China	P	Plasm	CLEIA	41 pg/ml	52.9	NA	Sepsis	68	LI	68	136	60	10	8	58	88.4	85.2	0.882
Lei (22)	Adults	China	P	Plasm	CLEIA	30.16 pg/ml	54.5	NA	Sepsis	110	SIRS	110	220	75	24	35	86	68.34	78.09	0.742

P, prospective study; R, retrospective study; CLEIA, chemiluminescent enzymeimmunoassay; TP, true positive; FN, false negative; FP, false positive; TN, true negative; SIRS, systemic inflammatory response syndrome; NA, not available; HC, healthy controls; LI, local infections.

### Quality assessment in each study

**FIGURE 2:**
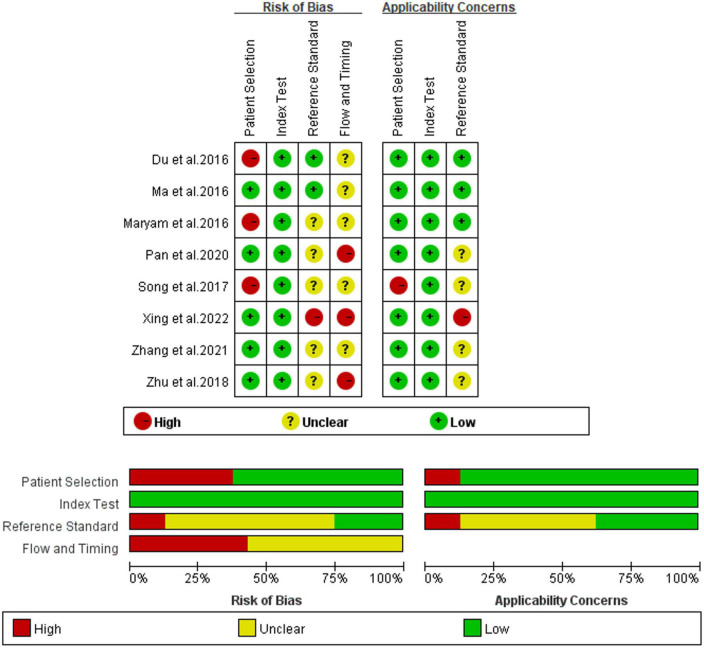
Bias risks and applicability concerns of included research.

Utilizing the QUADAS-2 method, the quality of eight studies on IL-35 for differential diagnosis of septic patients was assessed. Figure 2 displays the findings in detail, as seen here. In at least one category, every study posed an uncertain or significant risk of bias. Three investigations (16, 17, 19) revealed ambiguous or high-risk patient selection bias, mostly due to improper exclusion criteria and the lack of a clear definition for exclusion criteria. Five studies (17, 18, 20–22), showed significant or ambiguous applicability problems in relation to the reference standard.

### Publication biases

**FIGURE 3:**
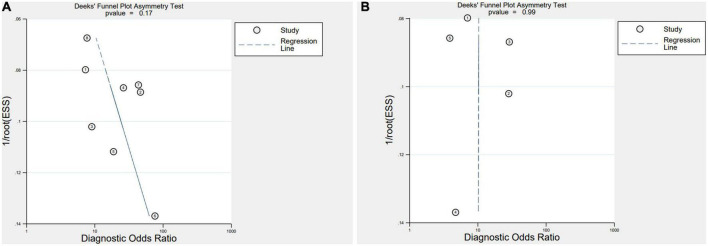
Deeks’ funnel plot **(A)**, interleukin 35 (IL-35) **(B)**, procalcitonin (PCT).

Deeks’ Funnel Plot Asymmetry Test revealed no indication of publication bias (IL-35: *P* = 0.17, PCT: *P* = 0.99) (Figure 3).

### Outcomes

**FIGURE 4:**
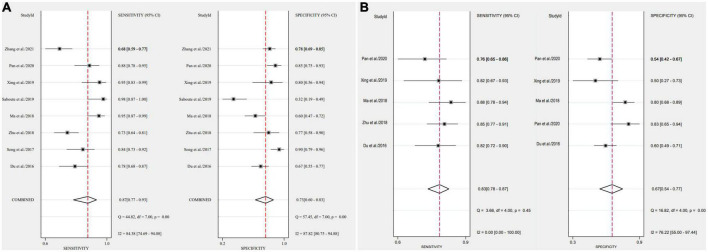
Forest plots of pooled sensitivity and specificity of biomarkers aimed at sepsis **(A)**, interleukin 35 (IL-35) **(B)**, procalcitonin (PCT).

**FIGURE 5:**
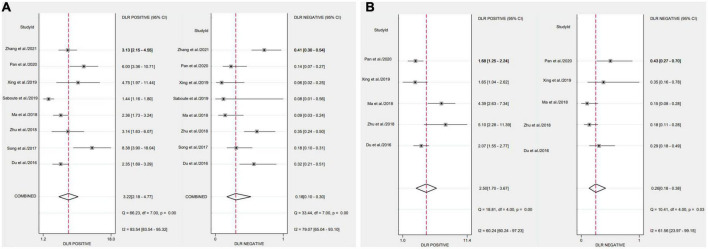
Forest plots of likelihood ratio of biomarkers aimed at sepsis **(A)**, interleukin 35 (IL-35) **(B)**, procalcitonin (PCT).

**FIGURE 6:**
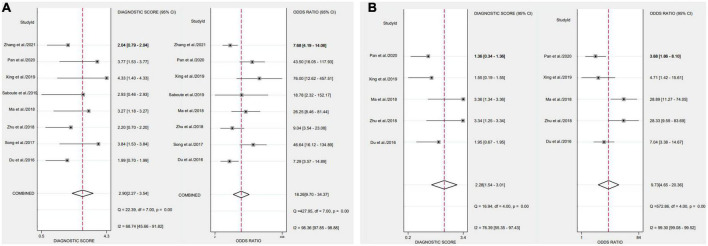
Forest plots of the diagnostic odds ratio of biomarkers aimed at sepsis **(A)**, interleukin 35 (IL-35) **(B)**, procalcitonin (PCT).

**FIGURE 7:**
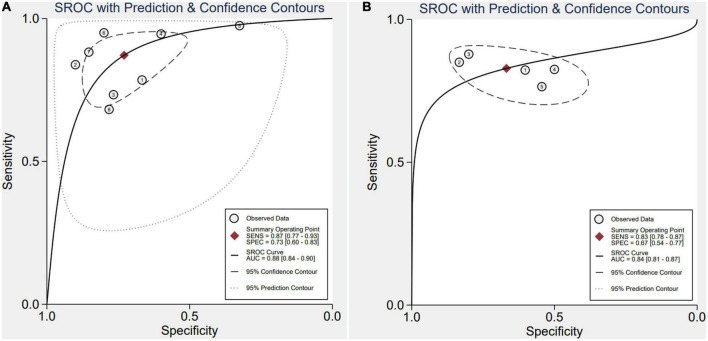
Symmetrical summary receiver operator curve (SROC) in all eight studies **(A)**, interleukin 35 (IL-35) **(B)**, procalcitonin (PCT).

After applying Stata 17.0 software in calculating the following results for IL-35: sensitivity 0.87 (95% CI, 0.77–0.93), specificity 0.73 (95% CI, 0.60–0.83) (Figure 4A), DOR 18.26 (95% CI, 9.70–34.37), PLR 3.22 (95% CI, 2.18–4.77), NLR 0.18 (95% CI, 0.10–0.30) and the DOR was 2.90 (95% CI 2.27–3.54) (Figures 5A, 6A). 0.88 (95% CI, 0.84–0.90) was the combined AUC (Figure 7A), which indicates that IL-35 is a pinpoint accurate biomarker in the diagnosis of sepsis. Results for PCT: sensitivity 0.83 (95% CI, 0.78–0.87), specificity 0.67 (95% CI, 0.54–0.77) (Figure 4B), DOR 9.73 (95% CI, 4.65–20.36), PLR 2.50 (95% CI, 1.70–3.67), NLR 0.26 (95% CI, 0.18–0.38) and the DOR was 2.28 (95%CI 1.54–3.01) (Figures 5B, 6B). 0.84 (95% CI, 0.81–0.87) was the combined AUC (Figure 7B).

### Fagan plot analysis

**FIGURE 8:**
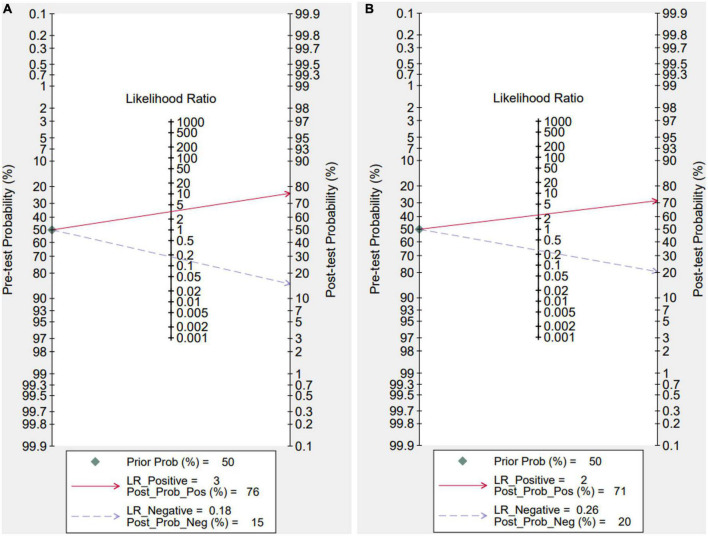
Fagan nomogram for interleukin 35 (IL-35) in sepsis diagnosis **(A)**, IL-35 **(B)**, procalcitonin (PCT).

To further investigate the potential value of IL-35 and PCT for sepsis diagnosis in the therapeutic setting, we built a Fagan nomogram, which revealed that detected IL-35 and PCT could improve the post-probability to 76%, 71%, and decrease the post-probability to 15%, 20%, both with a pre-test probability of 50%. Our findings suggest that IL-35 is a more clinically beneficial sepsis diagnostic tool than PCT (Figure 8).

### Analysis of heterogeneity

We conducted an analysis to establish the degree of dissimilarity between each of the eight research. In this meta-analysis of IL-35 and PCT, the *P*-value for the threshold analysis were 0.262, -0.6, and 0.531, 0.285, showing there was no threshold effect. In addition, the sensitivity of *I*^2^ was 84.38 (95% CI, 74.69–94.08), and the specificity was 87.82 (95% CI, 80.75–94.88) for IL-35, whereas the sensitivity of *I*^2^ was 0 (95% CI, 0–100) and the specificity was 76.22 (95% CI, 55–97.44) for IL-35, with significant heterogeneity among the studies.

**FIGURE 9:**
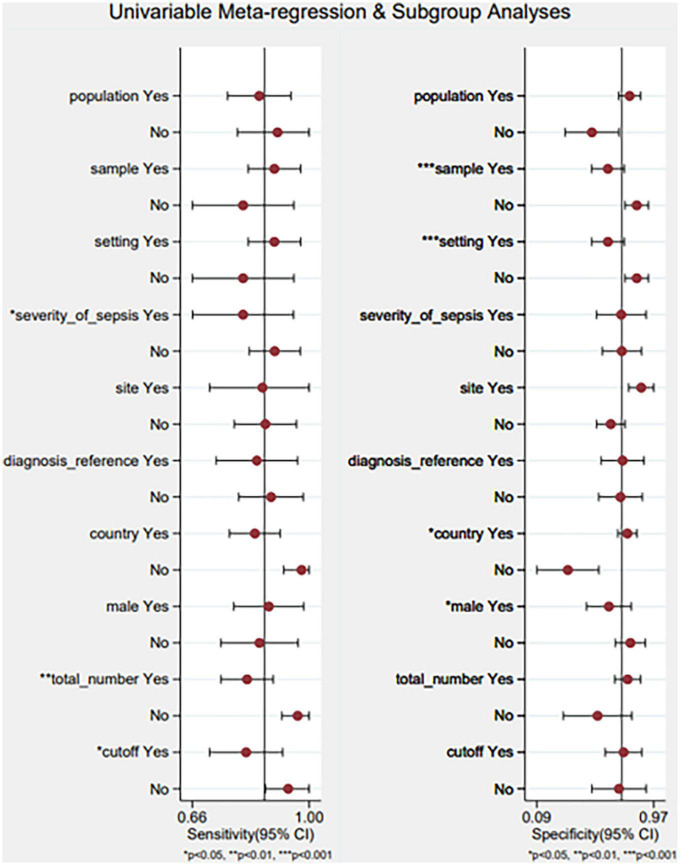
Meta-regression using a single variable and subgroup analysis. Yes/no represents that: population (adults/neonates), sample (serum/plasm), setting (ICU/other), severity of sepsis (severe sepsis or septic shock/other), site (celiac infection/other), diagnostic reference (sepsis-3/other), country (China and Iran), male (%) (≥55/<55), total number of samples (≥100/<100), cutoff (≥50/<50 pg/ml).

We built a meta-regression using a single variable and subgroup analysis to identify the causes of heterogeneity in the population, sample, setting, severity of sepsis, site, diagnostic reference, country, male (%), and the total number of samples (Figure 9). The results indicated that the sample, setting, severity of sepsis, country, male (%), the total number of samples, and cutoff values could account for the heterogeneity. The findings are shown in Supplementary Table 1.

## Discussion

Sepsis is still a worldwide health problem. Despite recent advances in treatment and diagnosis, the mortality rate of sepsis and septic shock in hospitalized patients continues to exceed 30 percent (25). For a long time, the pathology of sepsis has been regarded to be made up of the complex interplay between pro and anti-inflammation, and new early biomarkers are critically required since sepsis-related bad outcomes increase with each hour of delayed management (26). Various biomarkers, such as PCT, C-reactive protein, and soluble triggering receptor expressed on myeloid cells-1, are being used (by themselves or in conjunction) in the identification of sepsis. However, the clinical use of these indicators remains debatable (27). In addition, the gold standard for determining if a person has sepsis is blood culture, however, 48–72 h are required to achieve a result using this method, and due to its low positive rate, diagnosis is delayed and prevents optimal therapy from being administered. Therefore, it is crucial to identify a valid biomarker for the early and quick detection of sepsis (28).

Interleukin 35 is composed of the p35 component of IL-12 and Epstein–Barr virus-induced gene 3 (EBI3). In addition, IL-35 signaling is conducted by either separate heterodimers of gp130 and IL-12Rb2 or homodimers of these molecules. Recent research revealed that placental macrophages, dendritic cells, and trophoblasts express p35 and EBI3 (14). IL-35, secreted by plasma cells (CD138^+^ CD38^+^ subpopulation), can treat periodontitis by balancing the states between the host immune system and oral microorganisms in human studies. IL-35 exerts important regulatory activities, so there are various investigations of IL-35 expression in serum collected from septic patients. These results of the current meta-analysis suggest that due to its moderate sensitivity (0.87) specificity (0.73), and AUC (0.88), IL-35 can be a promising biomarker for sepsis than PCT with sensitivity (0.83) specificity (0.67), and AUC (0.84). Its diagnostic performance is superior to that of the conventional inflammatory biomarker, PCT, with higher AUC, sensitivity and specificity demonstrating its applicability. Therefore, we recommend measuring serum IL-35 as an inspection test for individuals clinically suspecting sepsis. This is in addition to further laboratory testing and sepsis signs. This screening method is risk-free, does not entail invasive procedures, is simple, and is affordably priced.

This is the first meta-analysis of its sort to evaluate the role of IL-35 in the diagnosis of sepsis. In addition, Fagan’ s nomogram is constructed, which is a tool that may be utilized in clinical practice to immediately assess the post-test probability. To further investigate the potential benefit of IL-35 for sepsis diagnosis in the profession of medicine, a Fagan nomogram was made by us, which revealed that detected IL-35 might enhance the post-probability to 76% and decrease it to 15%, assuming a 50% pre-test probability. The results reveal that IL-35 has a high diagnostic accuracy for sepsis, indicating that it may be a promising candidate for usage as a biomarker for the illness.

Different experiments were conducted and the possible reasons for this heterogeneity include regional differences, kit differences, and so on for each study, which explains why the cut-off values vary. In order to identify sepsis, a low threshold value, which results in identical sensitivity and specificity, could result in false-positive findings; consequently, it is more important to utilize high cut-off values. In the majority of the included investigations, cut-offs were determined *post-hoc* using ROC and Youden’s indices. Youden’s index has the benefit of having a single measurement, but it does not distinguish between a test’s sensitivity and specificity. As are other error-based metrics, such as the AUC, an indicator of a test’s overall precision. Defining the cutoffs after obtaining the data decreases the possibility that another investigation will duplicate the findings (29).

The inclusion criteria, sepsis definitions, and positive thresholds varied significantly across studies, making it impossible for us to officially analyze all of these factors as potential causes of variability. Further research into the literature included for this meta-analysis revealed substantial statistical heterogeneity, which may be related to sample, setting, the severity of sepsis, country, male (%), the total number of samples and cutoff values, etc.

We should be aware of some flaws in our meta-analysis that has to be discussed. To begin with, due to our strict inclusion criteria, we only included eight papers, so our research may lack the sufficient statistical ability to draw a clear conclusion. More clinical diagnostic studies are required to draw a conclusion. Second, we found significant heterogeneity in the included studies. Further research should attach importance to this problem. Third, the sample size of some included studies is small. Also, this meta-analysis only considered IL-35 as one of the diagnostic markers of sepsis and did not compare IL-35 with other diagnostic markers of sepsis.

However, in order to establish a conclusive assessment of its therapeutic use, various aspects must be explored. Future prospective studies on a large scale will be able to evaluate how reliable IL-35 is in the diagnosis and classification of sepsis. In addition, serum IL-35 must be tested routinely during the duration of medication in order to better comprehend its role in disease monitoring. In conclusion, IL-35 levels should be evaluated with clinical factors and other laboratory tests in order to develop a combined model with the maximum potential diagnostic performance. Future studies would benefit from the development of a superior consensus-building method for the assessment of diagnostic indicators that includes research design, the choice of patients, sample time, positive thresholds, Definitions of sepsis, and reporting of results.

## Conclusion

In conclusion, our results suggest that IL-35 can be applied as a candidate diagnostic biomarker for sepsis. The clinical interpretation of IL-35 results should also consider other traditional markers and the diverse clinical backgrounds of individual patients. To corroborate the findings of our study, other studies with significant participant populations must be done.

## Data availability statement

The original contributions presented in this study are included in the article/Supplementary material, further inquiries can be directed to the corresponding author.

## Author contributions

YH and DT designed and performed the study and wrote the manuscript. PZ was responsible for the revision and review of the manuscript. YH provided the ideas of the manuscript. YH and PZ were responsible for collecting the data. All authors contributed to the article and approved the submitted version.

## Supplementary material

Supplementary Table 1 Summary of meta-regression analysis.
