# Supplementary material for: The diagnostic value of interleukin 35 as a septic biomarker: A meta-analysis
Source: Front Med (Lausanne). 2022 Nov 10;9:999892. doi: 10.3389/fmed.2022.999892 (PMC9691458; doi:10.3389/fmed.2022.999892)
Supplement: Supplementary Table 1 — Summary of meta-regression analysis. [file Table_1.DOCX]

| Parameter | category | nstudies | Sensitivity | p1 | Specificity | p2 |
| --- | --- | --- | --- | --- | --- | --- |
| population | yes | 6 | 0.86 [0.77 - 0.95] | 0.29 | 0.79 [0.71 - 0.87] | 0.2 |
|  | no | 2 | 0.91 [0.79 - 1.00] | . | 0.50 [0.30 - 0.71] | . |
| sample | yes | 5 | 0.90 [0.82 - 0.98] | 0.96 | 0.63 [0.50 - 0.75] | 0 |
|  | no | 3 | 0.81 [0.66 - 0.96] | . | 0.84 [0.76 - 0.93] | . |
| setting | yes | 5 | 0.90 [0.82 - 0.98] | 0.96 | 0.63 [0.50 - 0.75] | 0 |
|  | no | 3 | 0.81 [0.66 - 0.96] | . | 0.84 [0.76 - 0.93] | . |
| severity of sepsis | yes | 3 | 0.81 [0.66 - 0.96] | 0.04 | 0.73 [0.54 - 0.91] | 0.52 |
|  | no | 5 | 0.90 [0.83 - 0.97] | . | 0.73 [0.58 - 0.88] | . |
| site | yes | 2 | 0.87 [0.71 - 1.00] | 0.55 | 0.88 [0.78 - 0.97] | 0.44 |
|  | no | 6 | 0.87 [0.78 - 0.96] | . | 0.65 [0.54 - 0.76] | . |
| diagnosis reference | yes | 4 | 0.85 [0.73 - 0.97] | 0.17 | 0.74 [0.57 - 0.90] | 0.58 |
|  | no | 4 | 0.89 [0.80 - 0.98] | . | 0.72 [0.56 - 0.89] | . |
| country | yes | 7 | 0.84 [0.77 - 0.92] | 0.18 | 0.77 [0.70 - 0.84] | 0.01 |
|  | no | 1 | 0.98 [0.93 - 1.00] | . | 0.32 [0.09 - 0.56] | . |
| male | yes | 4 | 0.88 [0.78 - 0.98] | 0.55 | 0.63 [0.46 - 0.80] | 0.04 |
|  | no | 4 | 0.86 [0.75 - 0.97] | . | 0.80 [0.68 - 0.91] | . |
| total number | yes | 6 | 0.82 [0.75 - 0.90] | 0 | 0.77 [0.68 - 0.87] | 0.36 |
|  | no | 2 | 0.97 [0.92 - 1.00] | . | 0.55 [0.29 - 0.81] | . |
| cut off | yes | 5 | 0.82 [0.71 - 0.92] | 0.01 | 0.74 [0.61 - 0.88] | 0.73 |
|  | no | 3 | 0.94 [0.87 - 1.00] | . | 0.71 [0.50 - 0.91] | . |
